# Treatment with metformin in twelve patients with Lafora disease

**DOI:** 10.1186/s13023-019-1132-3

**Published:** 2019-06-21

**Authors:** Francesca Bisulli, Lorenzo Muccioli, Giuseppe d’Orsi, Laura Canafoglia, Elena Freri, Laura Licchetta, Barbara Mostacci, Patrizia Riguzzi, Federica Pondrelli, Carlo Avolio, Tommaso Martino, Roberto Michelucci, Paolo Tinuper

**Affiliations:** 1 IRCCS Istituto delle Scienze Neurologiche di Bologna, Ospedale Bellaria, Bologna, Italy; 2 Department of Biomedical and Neuromotor Sciences, University of Bologna, Bologna, Italy; 3 Epilepsy Centre, Clinic of Nervous System Diseases, University of Foggia, Ospedali Riuniti, Foggia, Italy; 4 Department of Neurophysiology and Diagnostic Epileptology, Fondazione IRCCS Istituto Neurologico Carlo Besta, Milan, Italy; 5 Pediatric Neurology, Fondazione IRCCS Istituto Neurologico Carlo Besta, Milan, Italy

**Keywords:** Metformin, Lafora disease, Progressive myoclonus epilepsy, *EPM2A*, *EPM2B*, *NHLRC1*

## Abstract

**Background:**

Lafora disease (LD) is a rare, lethal, progressive myoclonus epilepsy for which no targeted therapy is currently available. Studies on a mouse model of LD showed a good response to metformin, a drug with a well known neuroprotective effect. For this reason, in 2016, the European Medicines Agency granted orphan designation to metformin for the treatment of LD. However, no clinical data is available thus far.

**Methods:**

We retrospectively collected data on LD patients treated with metformin referred to three Italian epilepsy centres.

**Results:**

Twelve patients with genetically confirmed LD (6 *EPM2A*, 6 *NHLRC1*) at middle/late stages of disease were treated with add-on metformin for a mean period of 18 months (range: 6–36). Metformin was titrated to a mean maintenance dose of 1167 mg/day (range: 500–2000 mg). In four patients dosing was limited by gastrointestinal side-effects. No serious adverse events occurred. Three patients had a clinical response, which was temporary in two, characterized by a reduction of seizure frequency and global clinical improvement.

**Conclusions:**

Metformin was overall safe in our small cohort of LD patients. Even though the clinical outcome was poor, this may be related to the advanced stage of disease in our cases and we cannot exclude a role of metformin in slowing down LD progression. Therefore, on the grounds of the preclinical data, we believe that treatment with metformin may be attempted as early as possible in the course of LD.

## Introduction

Lafora disease (LD) is a lethal, autosomal recessive, progressive myoclonus epilepsy. LD is caused by mutations in *EPM2A* or *NHLRC1*, encoding laforin and malin, respectively. With loss of function of either, structurally abnormal glycogen becomes insoluble and accumulates as Lafora bodies, responsible for disease progression [1]. Disruptions in cell homeostasis such as proteasomal dysfunction, oxidative stress, autophagy impairment, and mitochondrial dysfunction, also play a role in the pathophysiology of LD [2]. Symptoms typically begin in adolescence, and death commonly occurs within 10 years of onset. Antiepileptic drugs (AED) are partially effective on myoclonus and seizures but don’t have a major influence on the progression of cognitive and behavioural symptoms [3]. Despite the presence of new promising treatment strategies, no targeted therapy for LD in humans is currently available [3]. Metformin is an activator of AMP-induced kinase (AMPK) and is the most commonly prescribed drug for type 2 diabetes mellitus [4, 5]. AMPK is a key cellular energy sensor that, once activated by falling energy status, responds by activating catabolic pathways and inhibiting anabolic ones, such as glycogen synthesis [6]. Interestingly, the reduction of brain glycogen synthesis is one of the most promising therapeutic avenues for LD [3]. Moreover, through the activation of AMPK, metformin promotes autophagy and can also prevent brain mitochondrial dysfunction, decrease oxidative stress, and inhibit apoptotic cascade by preventing the permeability transition pore opening [7–11]. Through these and possibly other mechanisms, metformin acts as a neuroprotective agent in different neurodegenerative diseases [11–14]. It was also shown to facilitate seizure termination in mice [15]. A mouse model of LD treated with metformin showed amelioration of neuropathological symptoms, reduced seizure susceptibility and decreased accumulation of Lafora bodies [16, 17]. In 2016, the European Medicines Agency granted orphan designation to metformin for the treatment of LD (decision number EU/3/16/1803). However, no clinical data on treatment with metformin in LD is available thus far [3], also because of its extreme rarity. Here, we present a case series of LD patients treated with metformin.

## Methods

We collected data from three Italian epilepsy centres in which treatment with add-on metformin was proposed to all referred patients with genetically confirmed LD, irrespective of the stage of disease progression. Liver and kidney dysfunction, which may predispose to the development of lactic acidosis in diabetic patients treated with metformin, were excluded by standard laboratory testing in all patients. The stage of disease progression was assessed using a disability scale based on the residual motor and mental functions, daily living and social abilities [18]. Metformin was titrated to an individual target dose starting from 500 mg/die, depending on tolerability and clinical response. The latter was assessed considering the frequency of generalized tonic-clonic seizures, myoclonus severity, as well as the clinical, patient, and caregiver global impression.

Patients were evaluated at least at monthly frequency, either on an outpatient basis, during hospitalisation, or using telephone interviews with caregivers and home-made videos. If patients were hospitalised in institutes other than the three participating centres, data on their clinical status were obtained through the referring clinicians.

## Results

### Study population and treatment details

Twelve consecutive Italian patients with genetically confirmed LD were screened for contraindications and were consequently treated with add-on metformin. The first patient commenced treatment in February 2016. Last follow-up visit was in March 2019. Two cases were previously reported [19]. Clinical features of the subjects and treatment details are summarized in Table 1.

**Table 1:** Clinical features and treatment details

Clinical Features				Pre-Treatment Assessment					Metformin Treatment			
Pt	Age (y), Sex	Onset age (y)	Mutation^a^	Disease duration (y)	Concomitant AEDs	No. GTCS (last 28 days)	Myoclonus^b^	Disability scale^c^	Maximal dose (mg), adjusted dose (mg)	Outcome	Duration (months)	Adverse events
1	17^d^, F	13	*EPM2A*: c.491 T > G (p.Ile164Ser), c.539 T > C (p.Leu180Pro)	4	VPA, ZNS, PB	0^e^	+++	4	3000, 1500	Mild improvement (see text)	6	Diarrhoea
2	21, M	16	*NHRLC1*: c.436G > A (p.Asp146Asn), c.1133 T > C (p.Leu378Pro)	4	VPA, LEV, PER^f^, CNZ, CLB	18	+	3	2000, 1000	Seizure-freedom, improved cognition, lasted for 6 months	9	Diarrhoea
3^g^	25, F	13	*EPM2A*: c.243_246del (p.Asp82Argfs*7)	10	VPA, LEV, ZNS, PB, CNZ	3	+++	4	500, 500	Disease progression	12^h^	None
4^g^	19, F	10	*NHLRC1*: c.205C > G (p.Pro69Ala); c.826_829dup (p.Ala277Aspfs*23)	7	VPA, LEV, ZNS, CNZ	6	+++	4	1000, 500	Disease progression	24	Asthenia
5	17,F	13	*NHLRC1*: c.205C > G (p.Pro69Ala)	3	VPA, LEV, PER, CNZ	8	+++	4	2000, 2000	Disease progression	13	None
6	23, M	14	*EPM2A*: c.721C > T (p.Arg241*)	7	LEV, TPM, ZNS	7	+++	4	2000, 2000	Disease progression	9^h^	Elevated CPK, muscle cramps
7	23, M	10	*EPM2A*: c.721C > T (p.Arg241*)	11	VPA, PB, PHT, PER, CNZ	11	+++	3	2000, 2000	Disease progression	28	None
8	18, M	14	*NHLRC1*: c.992del (p.Gly331Glufs*3)	2	VPA, LEV, PER	2	++	2	1700, 500	Disease progression	26	Diarrhoea
9	22, F	13	*NHLRC1*: c.992del (p.Gly331Glufs*3)	7	VPA, ZNS, PER	3	+++	4	1700, 500	Disease progression	26	Diarrhoea
10	50, F	18	*EPM2A*: c.721C > T (p.Arg241*), c.835G > A (p.Gly279Ser)	29	VPA, LEV, ZNS, CNZ	1	+++	4	1000, 1000	Mild improvement (see text)	36	None
11	19, F	11	*EPM2A*: c.323G > T (p.Arg108Leu)	7	VPA, PB, PER, CNZ	0.5	+++	4	1000, 1000	Disease progression	12	None
12	18, M	13	*NHLRC1*: c.436G > A (p.Asp146Asn), c.1133 T > C (p.Leu378Pro)	4	VPA, LEV, ZNS, PB	3	++	3	1500, 1500	Disease progression	12	None

Legend: *AED* antiepileptic drug, *CLB* clobazam, *CNZ* clonazepam, *GTCS* generalized tonic-clonic seizures, *LD* Lafora disease, *LEV* levetiracetam, *PB* phenobarbital, *PER* perampanel, *PHT* phenytoin, *TPM* topiramate, *VPA* valproate, *ZNS* zonisamide

^a^When only one variant is indicated, this is intended as homozygous. *EPM2A* RefSeq ID: NM_005670.4; *NHLRC1* RefSeq: NM_198586.2

^b^Myoclonus severity: +, mild myoclonus, i.e. does not interfere with activities of daily living; ++, moderate myoclonus, i.e. interferes with activities of daily living but not with deambulation; +++, severe myoclonus, i.e. interferes with deambulation

^c^Disability scale developed by Franceschetti et al. [18]: 1, mild cognitive and motor impairment, preserved daily living activities, and social interaction; 2, moderate mental decline, limitations in motor activities and limited social interaction; 3, severe mental and motor impairment, needing help in walking and regular assistance in daily activity, and poor social interaction; 4, patient weelchair-bound or bedridden, and no significant daily living activities or social interaction

^d^deceased

^e^the patient did not present any GTCS but had 4 prolonged myoclonic seizures in 28 days requiring benzodiazepine rescue medications

^f^perampanel was introduced at the same time as metformin

^g^previously published in Ref. [19]

^h^suspended

Of the 12 patients, 7 were female. Mean age at disease onset was 13 years. Metformin was introduced at middle/late stages of disease, after a mean of 8 years from onset. Treatment duration ranged from 6 to 36 months (mean = 18 months). Metformin was titrated to an individual target dose depending on tolerability and clinical response, up to 3000 mg/day. The mean maintenance dose was 1167 mg/day (range: 500-2000 mg).

### Clinical outcome

In 9 out of 12 patients, metformin did not produce any relevant clinical benefit. In the remainder, we observed a clinical improvement. When metformin was introduced, patient 1 was staying in a long-term care facility in a vegetative state, had subcontinuous myoclonic jerks, no spontaneous motor activity and weekly generalized myoclonic seizures, lasting more than 5 min if not treated with benzodiazepines. During the 6 months of metformine therapy, the patient showed reduction of myoclonic seizures frequency with consequent reduction of benzodiazepine rescue medications, as well as the appearance of eye response to vocal stimuli. A further clinical deterioration followed, and the patient died of tracheostomy-related late bleeding. Patient 2 had a transitory clinical response, lasted approximately 6 months, characterized by improvement in behaviour and cognition and seizure-freedom. However, perampanel (up to 6 mg/day) was introduced at the same time as metformin because of the rapidly progressing clinical deterioration, and may therefore be responsible for the clinical improvement. Patient 10 had a long disease progression and at time of metformin introduction was bedridden, severely cognitively impaired, had subcontinous myoclonic jerks and approximately one generalized tonic clonic seizure (GTCS) per month. Treatment resulted in a reduction of myoclonus intensity, freedom from GTCS and an improved responsiveness, maintained for the 36 months of follow-up, during which concomitant AED regimen was not modified.

### Adverse events

Adverse events (AEs) were reported in six patients. The most common was diarrhoea (*n* = 4), which subsided in all patients after dose adjustment. Patient 4 reported asthenia with metformin at 1000 mg, not clearly related to treatment. Patient 6 had muscle cramps and elevated CPK, resolved after discontinuation. Patient 3 was given a maximal dose of 500 mg because pre-treatment basal glycaemia was at the lower normal limit. In this case, metformin was suspended after 12 months because there was no clinical benefit. No serious AEs were reported in any patient.

## Discussion

### Safety and tolerability

To the best of our knowledge, this is the first documentation of metformin use in humans with LD. None of our patients experienced serious AEs. In one case, reversible side effects brought to discontinuation. Gastrointestinal side effects are a well known metformin AE, are usually transient and subside once the dose is adjusted or when administered with meals [4]. Therefore, metformin was overall well tolerated and safe in our small cohort of LD subjects.

### Efficacy and study limitations

Of twelve treated patients, three had a clinical response, which was temporary in two. However, it was difficult to assess the role played by metformin in patient 2, who was simultaneously started on perampanel. Even though the disease eventually progressed in all treated patients but one with end-stage disease, we cannot exclude that metformin may have the potential to slow down LD progression, as no prospective study on LD natural history is available for comparison. The considerable mean delay of 8 years between disease onset and metformin introduction may be a potential reason for its apparent low efficacy. It is likely that the mechanisms by which metformin may ameliorate disease course in LD, i.e. inhibition of glycogen synthesis, autophagy promotion, reduction of oxidative stress, maintenance of mitochondrial capacities, and inhibition of apoptosis, might be more incisive if treatment is commenced soon after disease onset. Theoretically, the neuroprotective action of metformin would be even more pronounced if treatment is started in the pre-symptomatic phase in genetically diagnosed siblings of LD patients, in whom neuronal degeneration has not been established yet. Indeed, in the pre-clinical studies in which the efficacy of metformin treatment in a mouse model of LD was established, the drug was administered when the mice were 3 months old, at the beginning of their neurological impairment [16, 17].

We acknowledge the methodological limitations of our study, which was retrospective, not randomized, not controlled, and involved a small number of subjects. These limitations are, however, intrinsically related to the rarity of LD, to the lack of effective alternative therapies and to the overall safety of metformin, which brought us to offer this opportunity to all eligible patients.

## Conclusions

Metformin was overall safe in our small cohort of LD patients at middle/late stages of disease. Even though the clinical outcome was poor, this may be related to the relatively advanced stage of disease in our cohort and we cannot exclude a role of metformin in slowing down LD progression. Therefore, on the grounds of the preclinical data, we believe that treatment with metformin may be attempted as early as possible in the course of LD. Efficacy of metformin in LD should be further evaluated in randomized controlled trials involving larger cohorts of patients.

## Data Availability

The datasets during and/or analysed during the current study are available from the corresponding author on reasonable request.
